# Blood meal analysis of *Anopheles* vectors of simian malaria based on laboratory and field studies

**DOI:** 10.1038/s41598-021-04106-w

**Published:** 2022-01-10

**Authors:** Nantha Kumar Jeyaprakasam, Van Lun Low, Jonathan Wee Kent Liew, Sandthya Pramasivan, Wan-Yusoff Wan-Sulaiman, Atiporn Saeung, Indra Vythilingam

**Affiliations:** 1 Department of Parasitology, Faculty of Medicine, Universiti Malaya, Kuala Lumpur, Malaysia; 2 Tropical Infectious Diseases Research and Education Centre (TIDREC), Universiti Malaya, Kuala Lumpur, Malaysia; 3 Environmental Health Institute, National Environment Agency, Singapore, Singapore; 4 Department of Parasitology, Faculty of Medicine, Center of Insect Vector Study, Chiang Mai University, Chiang Mai, Thailand

**Keywords:** Biological techniques, Ecology

## Abstract

Blood feeding and host-seeking behaviors of a mosquito play an imperative role in determining its vectorial capacity in transmitting pathogens. Unfortunately, limited information is available regarding blood feeding behavior of *Anopheles* species in Malaysia. Collection of resting *Anopheles* mosquitoes for blood meal analysis poses a great challenge especially for forest dwelling mosquitoes. Therefore, a laboratory-based study was conducted to evaluate the potential use of mosquitoes caught using human landing catch (HLC) for blood meal analysis, and subsequently to document blood feeding behavior of local *Anopheles* mosquitoes in Peninsular Malaysia. The laboratory-based experiment from this study revealed that mosquitoes caught using HLC had the potential to be used for blood meal analysis. Besides HLC, mosquitoes were also collected using manual aspirator and Mosquito Magnet. Overall, 47.4% of 321 field-caught *Anopheles* mosquitoes belonging to six species were positive for vertebrate host DNA in their blood meal. The most frequent blood meal source was human (45.9%) followed by wild boar (27.4%), dog (15.3%) and monkey (7.5%). Interestingly, only *Anopheles cracens* and *Anopheles introlatus* (Leucosphyrus Group) fed on monkey. This study further confirmed that members of the Leucosphyrus Group are the predominant vectors for knowlesi malaria transmission in Peninsular Malaysia mainly due to their simio-anthropophagic feeding behavior.

## Introduction

Many countries in Southeast Asia are progressing towards eliminating malaria by the year 2030. Although Malaysia had reported zero indigenous cases since 2018^1^, increasing zoonotic malaria cases due to *Plasmodium knowlesi* is alarming. Knowlesi malaria is currently being reported in countries where *Anopheles* vectors from the Leucosphyrus Group, as well as their simian hosts, are present^2^. One of the strategies to progress towards malaria elimination is vector surveillance and control which have been highlighted by WHO in their Global Technical Strategy^3^.

Vector surveillance which includes the spatial distribution and density of the mosquito vectors could provide essential information to better understand the dynamics of malaria transmission and facilitate appropriate decisions regarding interventions. Distribution of the competent vectors, as well as their vectorial capacity contribute substantially to malaria endemicity^4^. There are several factors determining the vectorial capacity of *Anopheles* mosquitoes to transmit *Plasmodium* parasites. These include extrinsic incubation period of the parasites, daily survival of the mosquito^5^, gonotrophic cycle and gonotrophic discordance of the mosquito^6^ as well as the essential key element—feeding and host-seeking behaviors of the mosquito^5^.

*Anopheles* mosquitoes usually exhibit a wide range of host preferences including avian, human, livestock and reptiles^7^. Female *Anopheles* mosquitoes require blood meal for the production and development of their eggs^8^. Indeed, the prevalence of malaria is highly influenced by mosquito’s host selection behaviors which can be studied using blood meal analysis^9^. Besides, understanding the blood meal preferences of the *Anopheles* mosquitoes in malaria endemic areas is crucial for malaria vector identification^10^. The animal hosts in malaria endemic area could also play an important role in maintaining the vector populations. Unfortunately, very limited information is available regarding blood-foraging behavior and host preference of *Anopheles* species especially from the Leucosphyrus Group which are involved in *P. knowlesi* transmission in Peninsular Malaysia^2^.

One of the factors that hinders study on the blood meal preference of *Anopheles* mosquitoes is the difficulty in collecting the resting mosquitoes. Most of the blood meal analyses are conducted using resting *Anopheles* mosquitoes engorged with blood^10–12^. Obtaining resting *Anopheles* mosquitoes belonging to the Leucosphyrus Group which are generally forest-dwelling mosquitoes^2^ has been challenging^13^. Besides, different species of *Anopheles* mosquitoes have different feeding behaviors where some feed closer to ground while some species show preference for higher canopies with tendency to rest at higher places^14^. Thus, finding resting *Anopheles* mosquitoes in a dense rainforest like in Malaysia can be immensely challenging.

A couple of studies in Malaysian Borneo had evaluated the usage of multiple resting collection tools in attempt to collect resting *Anopheles* mosquitoes^13,15^. These includes resting bucket trap, sticky resting bucket, Centers for Disease Control (CDC) backpack aspirator^13^, Prokopac aspirator, gravid traps and Biogents (BG) sentinel traps^15^. Unfortunately, out of thousands of mosquitoes collected, only a single resting *Anopheles* mosquito was successfully obtained from each study. Other methods available to collect non-resting mosquitoes for blood meal analysis such as CDC light traps are inefficient at collecting *Anopheles* mosquitoes from the Leucosphyrus Group compared to human landing catch (HLC)^16,17^.

Therefore, a series of laboratory tests were conducted to evaluate the potential use of mosquitoes caught by HLC for blood meal analysis. To test this hypothesis, the present study used laboratory strain *An. cracens* to evaluate the meal preference of the mosquito after the initial blood meal and also the time limit for blood meal detection through PCR assays. With the ability to use mosquitoes caught by HLC in blood meal analysis, this study aimed to document blood feeding behavior of *Anopheles* mosquitoes, vectors of zoonotic simian malaria in Peninsular Malaysia. A deeper understanding on the complex mosquito blood feeding behaviors is crucial to elucidate the transmission potential of mosquito populations in Peninsular Malaysia to generate better predictions of *P. knowlesi* and other simian malaria transmission in the future.

## Results

### *Anopheles cracens* meal preference after initial blood feeding

**Figure 1:**
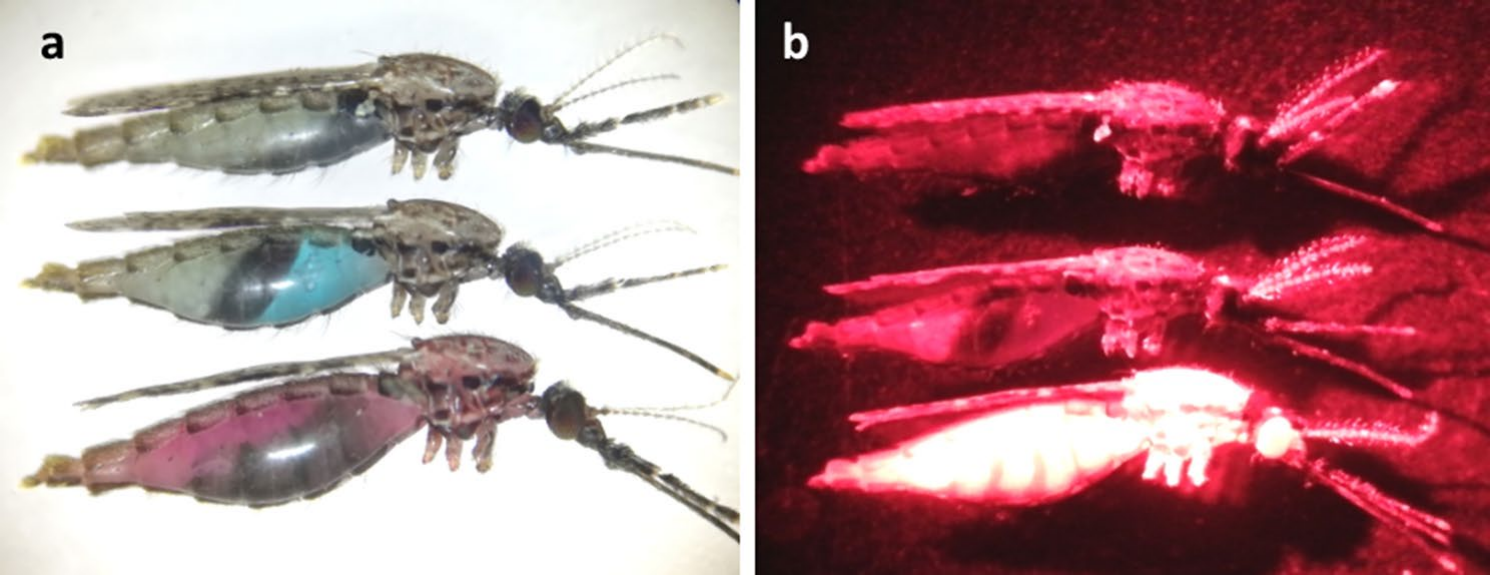
The image of *An. cracens* observed under the stereomicroscope (**a**) and fluorescent microscope (**b**). The mosquito on the top did not take any second meal; middle had taken sucrose solution infused with Brilliant blue dye while the bottom had taken second blood meal infused with rhodamine B fluorescent dye.

Mosquitoes which had taken second blood meal were easily detected using microscope. Mosquitoes which had taken the second blood meal infused with 0.1% rhodamine B, fluoresce in bright red under the fluorescent microscope while abdomen of mosquitoes which took sucrose solution infused with Brilliant blue dye appeared blue colour (Fig. 1).

**Figure 2:**
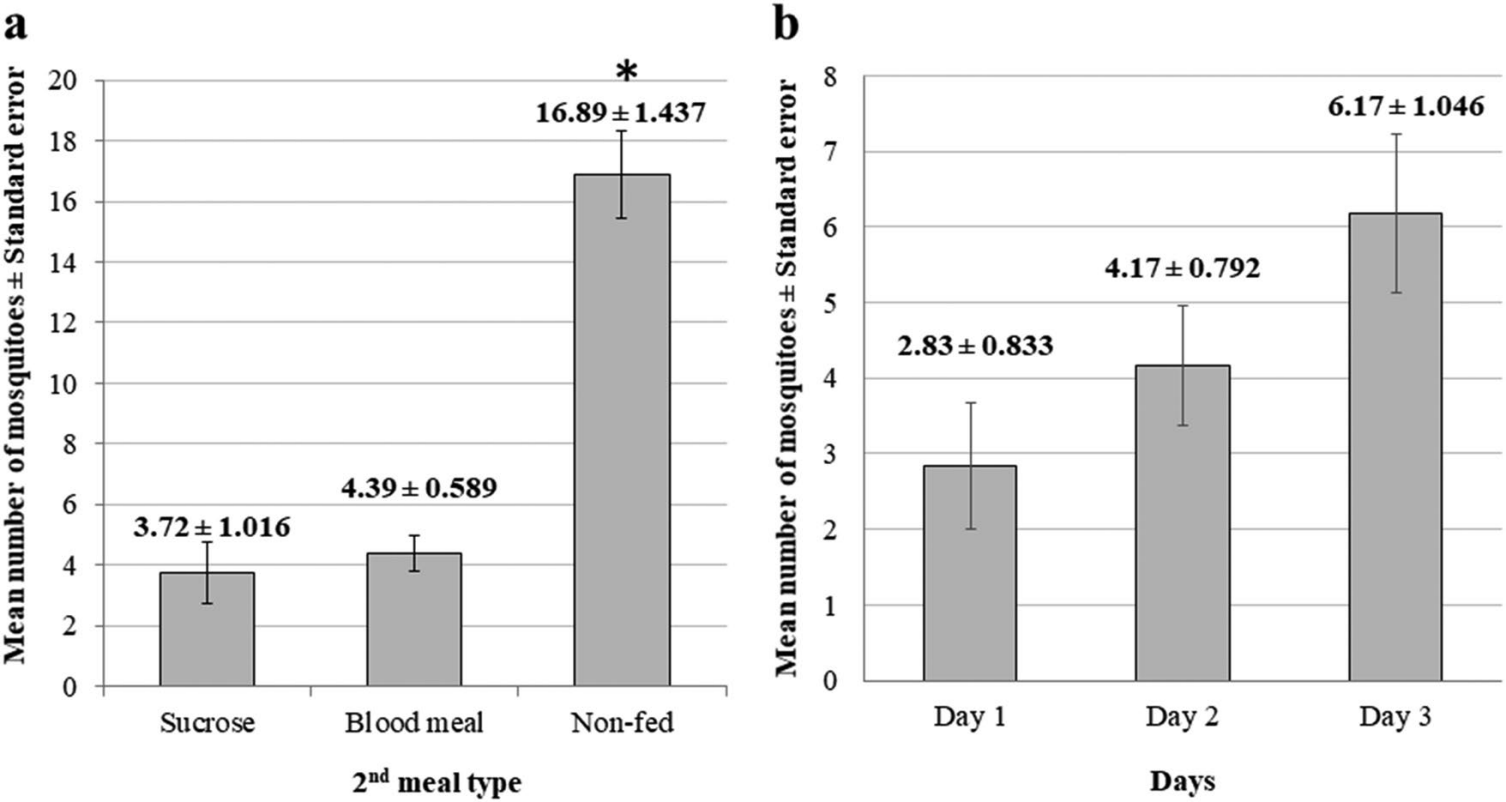
(**a**) The mean number (± standard error) of mosquitoes according to their second meal preference after initial human blood feeding within a single gonotrophic cycle. (**b**) The mean number (± standard error) of mosquitoes preferring second blood meal according to the number of days post-fed with human blood meal.

From the 450 *An. cracens* mosquitoes used in this experiment, 17.56% of the mosquitoes preferred a second blood meal within a single gonotrophic cycle while 14.89% preferred sucrose solution. A huge percentage of the mosquitoes (67.56%) went into resting position and did not take any meal after the initial blood feeding on Day 0. A Kruskal–Wallis test showed a significant difference between the types of meal preferred by the *An. cracens* mosquitoes within a single gonotrophic cycle after the initial blood feeding, *χ*^2^ = 30.850, *df* = 2, *P* < 0.001. Pairwise comparisons using Dunn’s test indicated that the mean number of mosquitoes which did not acquire a second meal (16.89 ± 1.437) was significantly higher (*P* < 0.001) than those fed on blood meal (4.39 ± 0.589) and sucrose (3.72 ± 1.016) (Fig. 2a). Nevertheless, the mean number of mosquitoes which preferred second blood meal was similar as those mosquitoes which preferred sucrose. The difference is not statistically significant (*P* = 0.849).

Although the mean number of mosquitoes which took the second blood meal was significantly lower than those did not feed, there was a steady increase in the mean number of mosquitoes taking second blood meal from day 1 until day 3 (Fig. 2b). A one-way repeated measure ANOVA was used to compare the number of mosquitoes taking a second blood meal for three different days from the initial blood feeding in a single gonotrophic cycle. Although there was an increase in the number of mosquitoes taking blood meal between these days, the increase was not statistically significant, *F* (2,10) = 1.128, *P* = 0.362, η^2^ = 0.184..

### Blood meal detection limit by PCR assay

**Figure 3:**
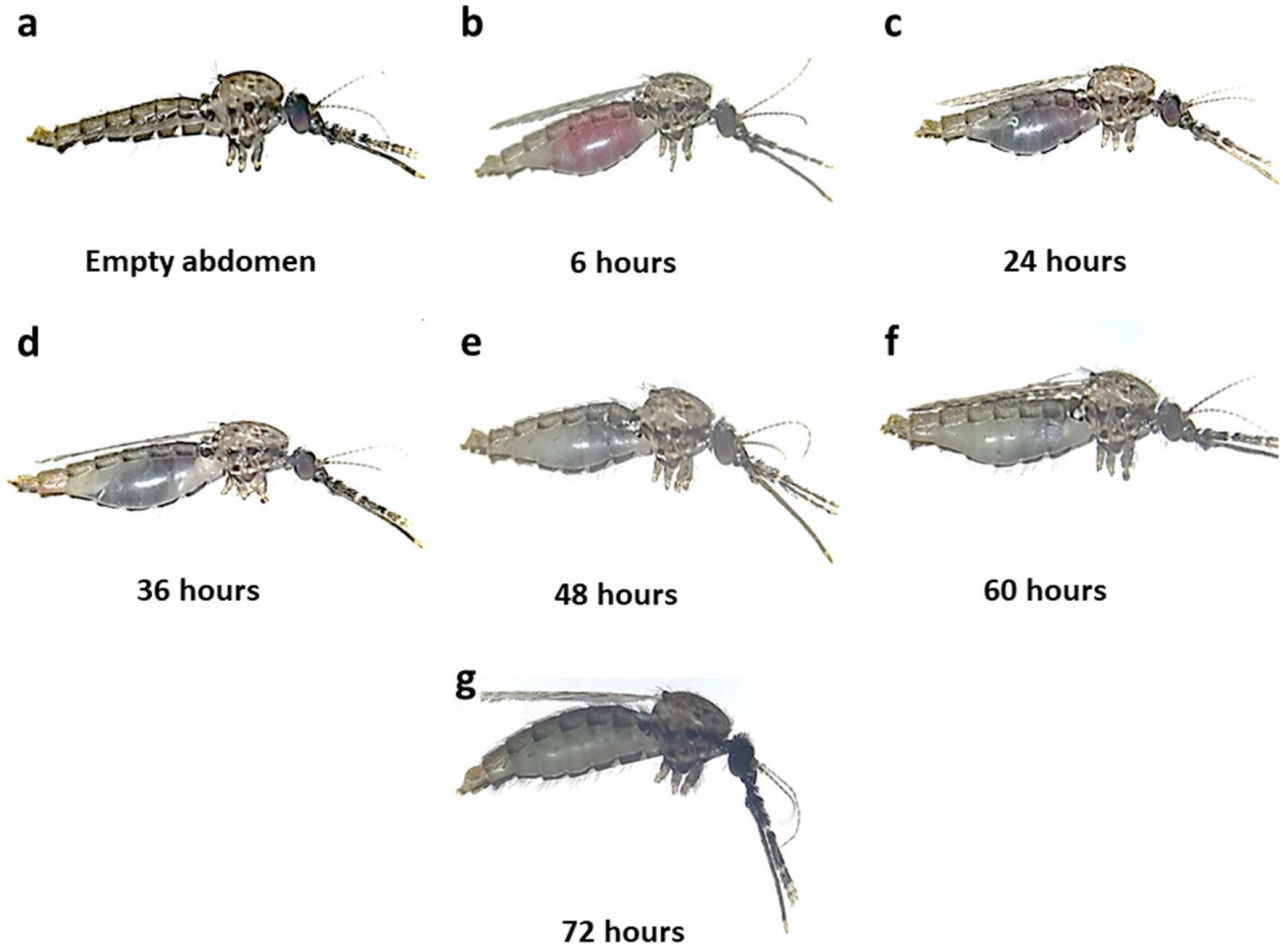
Females *Anopheles cracens* mosquitoes at different degrees of digestion based on the 7 stages of Sella scale: (**a**) Mosquito with empty stomach without blood and ovaries undeveloped; (**b**) Mosquito completely engorged with fresh blood that appeared bright red in colour with ovaries not developed at 6 h after blood meal; (**c**) Partially engorged where the anterior region of 5½–6 sternites and 3–4 tergites are occupied with dark blood at 24 h after blood meal; (**d**) Blood in the stomach appeared very dark which occupied the anterior region of 5–5½ sternites and 2–3 tergites at 36 h after blood meal; (**e**) The blood in the stomach appeared black and occupied the anterior region of 4½–5½ sternites and ½–1½ tergites at 48 h after blood meal; (**f**) The blood in the stomach appeared black and is only visible on the ventral side whereas the rest of the abdomen is filled with developing eggs at 60 h after blood meal; (**g**) Abdomen full with eggs with no visible blood at 72 h after blood meal.

The amplification success of the host DNA gradually decreased over the course of the digestion process. All mosquitoes were visually inspected, and the degree of digestion was classified according to the Sella scale^18^ (Fig. 3). Time course analysis showed that both human and monkey DNA were detectable in the mosquitoes’ abdomen up till 72 h post-feeding (Supplementary Fig. S1). However, as hours increased, the bands observed through gel electrophoresis appeared fainter, suggesting less intact DNA templates available for amplification due to the digestion process in the mosquitoes. There had been 100% success rate in amplifying both host’s DNA from 0 until 60 h post-feeding. However, at 72 h post-feeding, the percentage of human DNA (73.33%) successfully amplified from the mosquitoes was higher compared to monkey DNA (33.33%). Both host DNA failed to be amplified at 84 h and 96 h, suggesting that host DNA had been completely digested at these time points.

### Mixed blood meal analysis

Amplification of both host DNA (human and monkey) taken through two separate blood meals at different time point confirmed that multiple blood meal could be detected in a single gonotrophic cycle of a mosquito through the PCR assay. However, the amplified PCR products for the first blood meal (monkey blood) appeared less prominent in the gel electrophoresis compared to the second blood meal (human blood). This is likely due to the longer duration available to digest the first blood meal, thus lesser DNA template available for successful amplification (Supplementary Fig. S2). Nevertheless, the presence of faint bands for the monkey blood which was the first blood meal, indicates the ability of this PCR assay to detect multiple blood meals within a single gonotrophic cycle of a mosquito.

### Determination of blood meal origin of field caught *Anopheles* mosquitoes

The individual animal-specific PCR assays targeting the *COI* gene was used to amplify the different host bloods (Supplementary Fig. S3). Only seven common animals sighted at the sampling locations were included in the detection of the blood meal of the field-caught *Anopheles* mosquitoes. Overall, 321 *Anopheles* mosquitoes belonging to eight species: *An. aconitus* (n = 6), *An. barbirostris* complex (n = 4), *An. cracens* (n = 26), *An. introlatus* (n = 63), *An. kochi* (n = 2), *An. maculatus* (n = 148), *An. minimus* (n = 3) and *An. sinensis* (n = 69) were screened for the presence of DNA of the vertebrate host in their blood meal. Of these, 75.4% of the mosquitoes were caught using HLC, 21.5% through Mosquito Magnet (MM) while the remaining 3.1% were resting mosquitoes collected through manual aspirator. The resting mosquitoes were found near the shrubs and also on the tree trunks near a cow shed.

Since the laboratory-based experiment using *An. cracens* showed that the host DNA from the blood meal can be amplified up till 72 h post-feeding and a small percentage of *Anopheles* mosquitoes tended to take multiple blood meals within a single gonotrophic cycle, mosquitoes caught using HLC were also used in the blood meal analysis. The success rate in identifying blood meal was higher in the resting mosquitoes (80.0%) compared to mosquitoes collected through Mosquito Magnet (52.2%) and HLC (44.6%). The chi-square test showed significant difference in the success rate of identifying the source of blood meal of the mosquitoes among the different collection methods, χ^2^ = 11.627, *df* = 2, *P* = 0.003.

Nevertheless, mosquitoes from all the collection methods which were visually engorged with blood showed positive results when screened with vertebrate specific primers. Non-engorged mosquitoes were also screened for blood meal, since the laboratory experiment from this study revealed that mosquitoes even at stage 7 of the Sella scale can be detected for their blood meal, albeit with a relatively lower success rate. A higher percentage of mosquitoes caught using HLC had no visible blood in the abdomen (61.11%), but were positive when screened for presence of blood meal using PCR assay. Around 47.06% of mosquitoes collected by HLC with visible blood in the abdomen had a mixed blood meal of human and other animals, while 11.76% of the mosquitoes tested positive for DNA from a vertebrate host other than human, implying that *Anopheles* mosquitoes caught using HLC can be used in blood meal analysis to detect other animal bloods beside human (Supplementary Table S1). Irrespective of the method of mosquito collection, human DNA was present in higher percentage in the blood meal of the *Anopheles* mosquitoes; either singly or mixed with another vertebrate host.

Overall, from the 321 *Anopheles* mosquitoes collected, 152 mosquitoes were positive for the presence of vertebrate’s DNA (HLC = 108; MM = 36; resting mosquitoes = 8). The most frequent blood meal source was human (45.9%) followed by wild boar (27.4%), dog (15.3%), monkey (7.5%), bovine (2.1%) and cat (1.4%). On the other hand, only one mosquito had fed on chicken (0.4%) (Supplementary Table S2). All species of *Anopheles* mosquitoes fed on humans, with a very high average HBI of 0.85. The highest HBI was found in *An. sinensis* (0.90) followed by *An. maculatus* (0.87), *An. cracens* (0.80) and *An. introlatus* (0.76). HBI for *An. aconitus* and *An. barbirostris* complex was not calculated due to very low sample size. The DNA sequencing which was carried out for some of the randomly selected samples from each animal group confirmed the PCR results.

**Figure 4:**
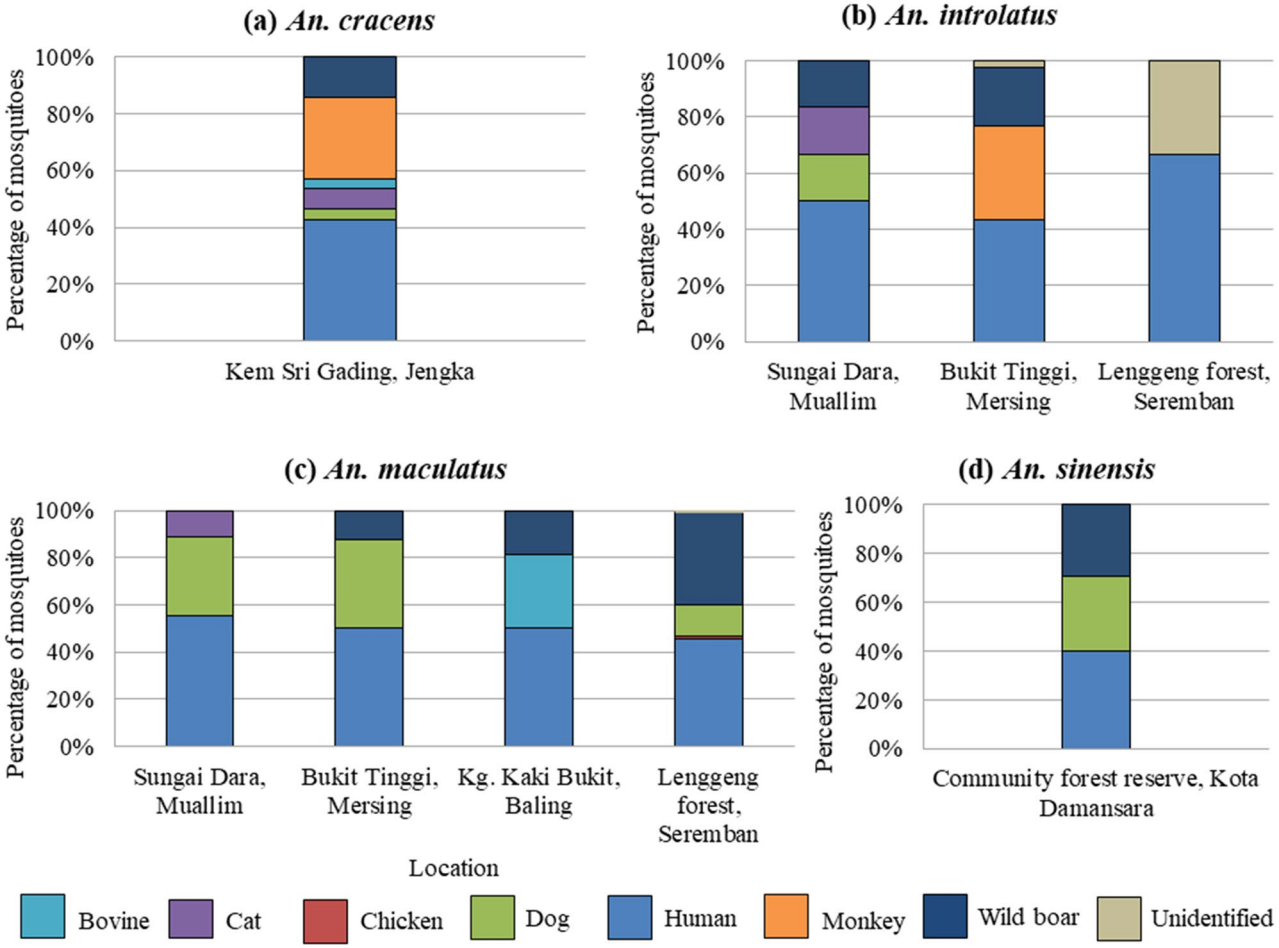
Source of blood meal of four predominant *Anopheles* species (**a**) *An. cracens*, (**b**) *An. introlatus*, (**c**) *An. maculatus* and (**d**) *An. sinensis* according to sampling locations.

*Anopheles cracens* and *An. maculatus* showed the widest range of blood meal sources (n = 6). This was followed by *An. introlatus* which had taken blood meal from five different vertebrate hosts. Three blood meal sources were detected from *An. sinensis* while two blood meals sources from *An. barbirostris* complex. On the other hand, only human blood was detected from *An. aconitus*. The blood meal preference of *Anopheles* mosquitoes was also analyzed according to geographical locations (Fig. 4). Both *An. barbirostris* complex and *An. aconitus* were excluded from the analysis since the number of the mosquitoes were very low. There were some differences in the proportion of host preference for the same species of *Anopheles* mosquitoes from different sampling locations. For example, the proportion of *An. maculatus* that fed on wild boar from Lenggeng forest, Seremban (39.0%) was higher compared to *An. maculatus* from Kg. Kaki Bukit, Baling (18.8%) and Bukit Tinggi, Mersing (12.5%). However, the most frequent blood meal sources for *An. maculatus* from all the sampling locations were human. For *An. sinensis*, human, wild boar and dog seem to be the most preferred sources of blood meal.

**Figure 5:**
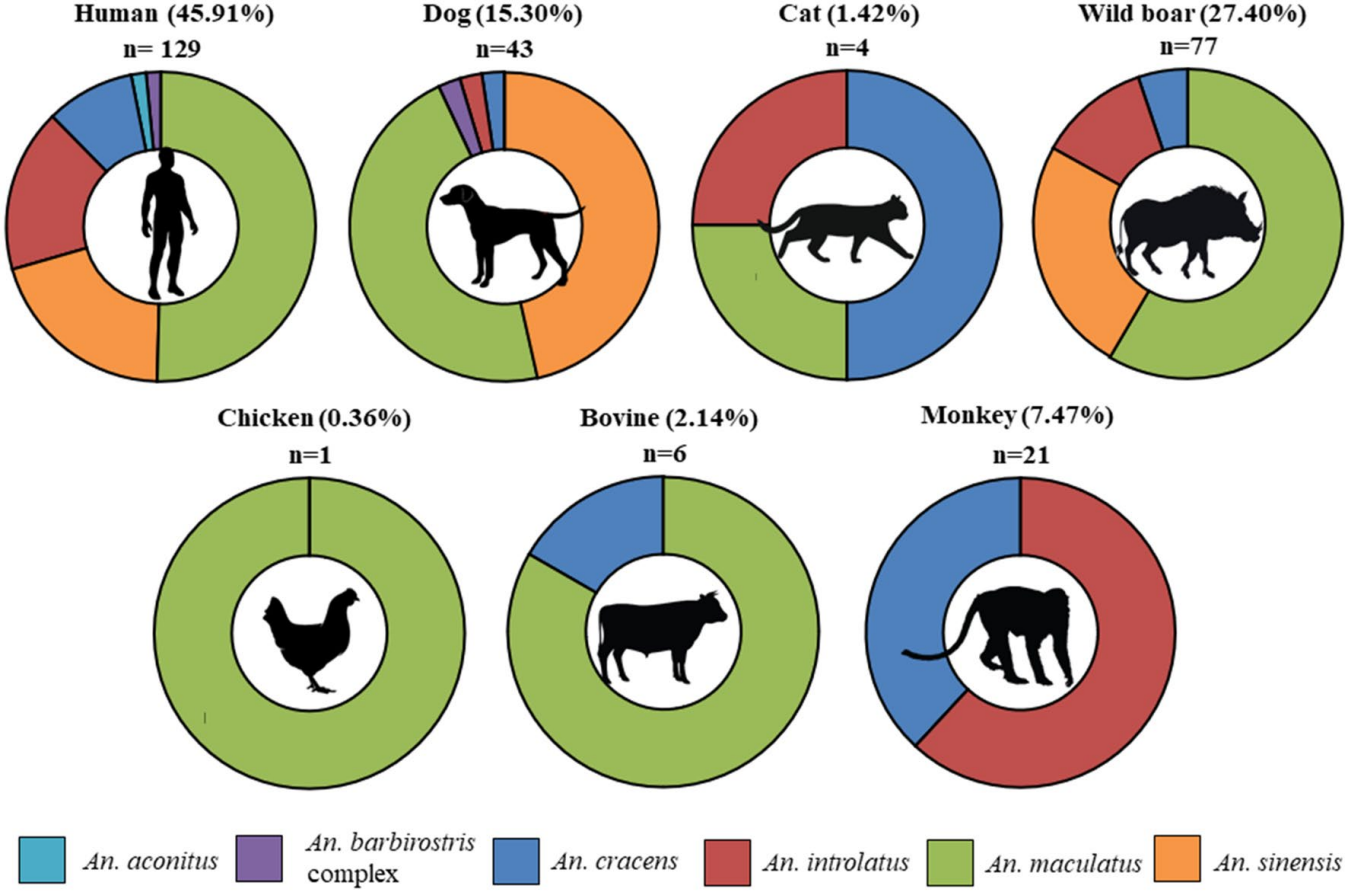
Proportion of host preference by *Anopheles* species.

In this study, only *An. cracens* and *An. introlatus* were from the Leucosphyrus Group. *Anopheles cracens* was only identified and collected from Kem Sri Gading, Jengka. They seemed to have higher preferences for human and monkey compared to other animals for their blood meal. On the other hand, higher number of *An. introlatus* was caught in Bukit Tinggi, Mersing. The *An. introlatus* caught from Sungai Dara, Muallim have slightly wider range of host preferences compared to *An. introlatus* from Bukit Tinggi, Mersing and Lenggeng forest, Seremban. Interestingly, from all the sampling locations, only *Anopheles* mosquitoes from the Leucosphyrus Group were positive for monkey in their blood meal analysis (Fig. 5).

Blood meal analysis also showed that most *Anopheles* species fed either from a single (31.6%) or two different animal sources (47.4%) (Table 1). Mosquitoes which fed on more than two different animals were also detected in a smaller proportion. The percentage of *Anopheles* fed on three different animals were 17.8% while interestingly 1.3% fed on four different animal sources. On the other hand, blood meal from three *Anopheles* mosquitoes which were positive for vertebrate DNA could not be identified to the species level suggesting the blood meal might have originated from other than the seven animals tested.

**Table 1:** Number of *Anopheles* mosquitoes with single and multiple blood meals.

	n	Single (n = 1)	%	Mixed (n = 2)	%	Mixed (n = 3)	%	Mixed (n = 4)	%	Unknown	%
*An. aconitus*	2	2	100.0	0	0.0	0	0.0	0	0.0	0	0.0
*An. barbirostris* complex	2	1	50.0	1	50.0	0	0.0	0	0.0	0	0.0
*An. cracens*	15	6	40.0	6	40.0	2	13.3	1	6.7	0	0.0
*An. introlatus*	29	12	41.4	12	41.4	2	6.9	1	3.4	2	6.9
*An. maculatus*	75	23	30.7	39	52.0	12	16.0	0	0.0	1	1.3
*An. sinensis*	29	4	13.8	14	48.3	11	37.9	0	0.0	0	0.0
Total	152	48	31.6	72	47.4	27	17.8	2	1.3	3	2.0

## Discussion

The transmission dynamic of mosquito-borne diseases such as malaria is highly dependent on the host preference and feeding behavior of the vector mosquitoes. Unfortunately, there is scanty data on blood meal analysis for *Anopheles* mosquitoes in Malaysia and to our knowledge there has been no blood meal study carried out for *Anopheles* mosquitoes in Peninsular Malaysia. One of the contributing factors might be due to the difficulties in finding resting *Anopheles* mosquitoes as demonstrated by studies in Sabah^13^ and Sarawak^15^, Malaysian Borneo. This indeed highlights the fundamental challenge in collecting resting *Anopheles* mosquitoes for blood meal analysis, especially the zoonotic malaria vectors. Although there are studies which had successfully collected resting *Anopheles* mosquitoes^9,10,19^, these were of different species from different geographical locations. Since most blood meal studies only utilized visually engorged resting mosquitoes, it became one of the major limitations which indirectly underestimates the proportion of host sources^10^.

There are many common collection methods available to catch resting mosquitoes. For examples, indoor resting mosquitoes can be collected through hand collection using aspirator, window trap collection (exit traps) or pyrethrum spray sheet collection^20^. On the other hand, outdoor-resting mosquitoes can be collected through backpack aspirators or pit shelters sampling methods^20^ though the yield is highly variable based on the sampling locations and mosquito species. In this study, the resting mosquitoes were only collected from Kampung Kaki Bukit, Baling Kedah, a village settlement near forest fringes, but none of the resting mosquitoes were from the Leucosphyrus Group. Unfortunately, there was no resting *Anopheles* mosquitoes collected from other sampling locations. In view of this, a series of laboratory tests was conducted to validate the use of mosquitoes caught using HLC for blood meal analysis. This is mainly because HLC was able to catch a higher number of local *Anopheles* mosquitoes, besides Mosquito Magnet^17^. Thus, this is the first study to evaluate the use of *Anopheles* mosquitoes collected through HLC for blood meal analysis. With this, our study aimed to document the hematophagic preference for *Anopheles* mosquitoes in Peninsular Malaysia with special focus on the vectors of simian malaria.

In the laboratory study, 17.56% of the *An. cracens* took a second blood meal within a single gonotrophic cycle. This indicates the probability of mosquitoes caught through HLC still have their previous blood meal in the abdomen which can be detected through PCR assay. Mosquitoes usually ingest blood meal to complete a gonotrophic cycle for successful reproduction. In many mosquitoes, one blood meal is required for the maturation of one batch of eggs^21^. Despite this, some culicines and anophelines exhibit gonotrophic discordance where they feed more than once per gonotrophic cycle^6,22^. Under laboratory conditions, some *Anopheles* mosquitoes such as *An. tessellates*^23^, *An. atroparvus*^24^, *An. albimanus*, *An. gambiae*^25^ and *An. stephensi*^26^ had been reported with gonotrophic discordance. The presence of mixed blood meal in many field captured *Anopheles* mosquitoes also further supports the possibility of gonotrophic discordance in wild *Anopheles* mosquitoes. The need for more than one blood meal to complete a single gonotrophic cycle is dependent on the nutritional content of the first ingested meal^27^, mosquito body size and metabolic reserve in the mosquito^28^. However, in our laboratory study, the percentage of *An. cracens* mosquitoes which had taken a second blood meal was significantly lower. Nevertheless, this idea paves the way for future investigation using other local *Anopheles* mosquitoes in Malaysia which are vectors for zoonotic malaria.

Although our laboratory study showed that the percentage of *An. cracens* taking second blood meal within a single gonotrophic cycle is lower, this percentage is hypothesized to be higher for the field caught mosquitoes. This is mainly because mosquitoes feeding in the wild were highly affected by the defensive behavior or the movement of the host which significantly reduces the feeding success. This eventually leads to multiple feeding behavior of the mosquitoes^27^. Contrarily, there is less disturbance in laboratory setting when the mosquitoes were blood-fed using hemotek membrane feeder since the environment was more controlled. Besides, environmental factors such as wind movement could also potentially disrupt the feeding process of the mosquitoes^29^, which leads to multiple blood feeding. This works in advantage for using mosquitoes caught by HLC where the previous blood meal can be analyzed to determine the host. This is in parallel with this study where nearly 65.8% of the *Anopheles* mosquito analyzed for blood meal showed mixed blood meal of two or more vertebrate hosts. The high number of mosquitoes with mixed blood meal from this study also suggests all those animals were living in close proximity to each other. Besides, the ability of the *Anopheles* mosquitoes to fly reasonable distance from the breeding sites also indicates higher possibilities for the mosquitoes to encounter a wide range of animals in the wilderness^30^. For example, *An. maculatus* can fly a distance approximately 1.2 km^31^ while *An. sinensis*, around 2 km^32^.

Laboratory-based experiment using *An. cracens* from this study also showed that the DNA of the blood meal was detectable until 72 h post-feeding through PCR assay. This further supports the idea of using mosquitoes caught by HLC for blood meal analysis. However, the rate of blood meal digestions could be influenced by many factors, where one of them is the species of the mosquitoes^33^. For example, the mean time for complete blood meal digestion at mean temperature of 25.5 °C for *An. gambiae* was 48 h while 60 h for *An. funestus*^34^ and around 80 h for *An. maculipennis*^18^. In this study, the digestion rate of *An. cracens* had been categorized based on the Sella scale which provides a simple and standardized visual representation to determine the stages of blood meal digestion within the mosquito. This is of interest since *An. cracens* is one of the important vectors for *P. knowlesi* in Peninsular Malaysia^35^. Indeed, the digestion of blood meal in a mosquito greatly reduces the ability to amplify the host DNA^19^. Thus, the Sella scale provides a clear and standardized visual tool of the seven stages of blood meal digestion and ovarian development within the mosquito which will be useful to determine the period over which a given molecular method will be effective in amplifying the host DNA.

The results from this study demonstrated that non-engorged mosquitoes can still be used for blood meal analysis although the success rate is gradually reduced over time due to digestion. This was showcased in this study, where a higher proportion of mosquitoes collected from HLC had no visible blood in their abdomen yet tested positive for blood meal using PCR assays. These mosquitoes could be at the final stage of digestion with no visible blood in their abdomen when examined through naked eye. However, when examined through stereomicroscope, the midgut sometimes has a slight tinge of blood. The laboratory experiment from this study indeed showed the ability of the PCR assay to detect blood meal at Sella stage 7 of the digestion of the mosquitoes, although the success rate is relatively lesser compared to earlier stages of digestion.

Among all the species of *Anopheles* mosquitoes from this study, *An. maculatus* and *An. sinensis* had the highest HBI. This was expected since both these mosquitoes are highly anthropophilic and vectors of human malaria in few countries in Southeast Asia^36^. However, *An. sinensis* is not a vector for malaria in Malaysia^37^. Contrarily, *An. maculatus* is the primary vector of human malaria in Peninsular Malaysia especially in the hilly regions^38^. Despite being regarded as anthropophilic, previous study using monkey baited traps showed a very small percentage (4.7%) of *An. maculatus* were attracted to monkeys^35^. However, in this study, none of the *An. maculatus* were positive for monkey blood; which might be due to the presence of other preferred hosts^5,39^. In this study, both *An. introlatus* and *An. cracens* showed a relatively high HBI. This is in parallel with previous studies which had highlighted the anthropophilic nature of both *An. introlatus*^38^ and *An. cracens*^35^ in Malaysia. However, the high HBI for these four species of *Anopheles* mosquitoes from this study might also be partially influenced by the method of catching the mosquitoes. Around 71% of the positive samples were caught using HLC where some of the mosquitoes might have already taken the blood from the volunteers before it was caught; which is one of the limitations of the study. However, extra cautions were taken not to include the mosquitoes which had obviously taken a blood meal from the volunteers. Indeed, the standard protocol for HLC is to catch the mosquitoes which landed on the bare legs before it bites. In addition, the PCR assay was still able to detect other vertebrate’s DNA besides human for mosquitoes caught by HLC. Although the success rate in identifying the blood meal from mosquitoes caught using HLC (44.6%) was significantly lower than in resting mosquitoes (80.0%), it still provided valuable information for blood meal analysis especially when identifying and collecting engorged resting mosquitoes is a momentous task in forested areas.

Our study showed that none of the *Anopheles* mosquitoes have a single preference for a specific host. This finding seems to support the idea that blood meal consumption is probably more driven by the host availability rather than host preference^10^. For example, only *An. maculatus* from Kg. Kaki Bukit, Baling Kedah was positive for bovine blood meal. It was because only at this sampling location cow was present out of three other locations where *An. maculatus* were collected. Thus, host preference by the *Anopheles* mosquitoes is indeed determined by multiple factors. Besides host availability, other factors which influence host preference for *Anopheles* mosquitoes include host density, host defense mechanism, host size, proximity to mosquito habitats^40^, environmental factors, flight behaviors and feeding periodicity of the mosquitoes^41^.

Interestingly, only *An. cracens* and *An. introlatus* were positive for monkey blood. Indeed, *An. cracens* was known to be simio-anthropophagic^35^. Both *An. cracens*^35^ and *An. introlatus*^38^ had been incriminated as the vector for *P. knowlesi* in Peninsular Malaysia. Although previously only *Anopheles* mosquitoes from the Leucosphyrus Group had been incriminated as the vector for *P. knowlesi*, recent studies had identified *An. donaldi* from the Barbirostris Subgroup^42^ and *An. collessi* and *An. roperi* from the Umbrosus Group^43^ as the new additional vector of *P. knowlesi* in Malaysian Borneo. Based on the blood-seeking preference of *An. cracens* and *An. introlatus* from our current study, *Anopheles* mosquitoes from the Leucosphyrus Group are still deemed to be the main vectors in the transmission of *P. knowlesi* in Peninsular Malaysia since only these two species fed on both human and monkey.

Nevertheless, it is necessary to conduct further studies involving larger sample size from various geographical locations for a greater understanding on the hematophagous behavior of the local *Anopheles* mosquitoes in Peninsular Malaysia. Indeed, blood-feeding patterns of mosquitoes are crucial for incriminating malaria vectors as it provides valuable information to understand pathogen transmission between different vertebrate groups^9^. Deforestation due to extensive development as well as land clearing for agriculture and human settlement brings human in close contact with the habitat of the macaques^44^. As a consequence, human is at risk of exposing themselves to emerging zoonotic simian malarias^45^, especially with the presence of competent vectors which have both anthropophilic and zoophilic behaviors as shown in this study. Mixed blood feeding behavior on human and macaques by the *Anopheles* mosquitoes collected from this study sites showed the inherent risk of pathogen transfers between the hosts which can eventually be a public health issue.

## Conclusion

The laboratory-based experiment from this study revealed that mosquitoes caught using HLC can still be used for blood meal analysis, especially when finding engorged resting mosquitoes in forested areas can be immensely challenging. Nevertheless, factors such as species of the mosquitoes should be considered since different species have different digestion rates. Besides, extra cautions are needed when interpreting the HBI value since methods of catching the mosquitoes do partially influences the value. The blood meal analysis from this study showed that *Anopheles* from the Leucosphyrus Group remained the main vector for knowlesi malaria transmission in Peninsular Malaysia mainly due to their simio-anthropophagic feeding behavior of the mosquitoes. However, the recent findings on the ability of *Anopheles* mosquitoes from the non-Leucosphyrus Group to transmit *P. knowlesi* in Borneo Malaysia underscore the importance for more intensive entomological studies on the local vectors. This includes the blood meal analysis where the data is very scarce in Malaysia. With the rapid change in the landscape and the potential emergence of other zoonotic simian malaria, blood meal analysis would be instrumental in monitoring the hematophagous behavior of the local *Anopheles* mosquitoes.

## Methods

### Experimental design

Laboratory based experiments were conducted in three parts to evaluate the potential use of mosquitoes caught using HLC from the field for blood meal analysis. Firstly, meal preference study was conducted to investigate the possibility of the *An. cracens* (laboratory strain) to uptake second blood meal within a single gonotrophic cycle. Secondly, the rate of blood digestion in the mosquito was observed through amplification of host DNA ingested by *An. cracens* through a time course of every 12 h for 96 h using PCR assay for both human and monkey blood. Finally, multiple blood feeding analysis was conducted using monkey and human blood to investigate if the PCR assay was able to detect multiple blood meals.

This PCR assay was later used to test the field caught *Anopheles* mosquitoes. Mosquitoes were collected between December 2019 and May 2021 at six different sampling locations within Peninsular Malaysia. The presence of domestic and wild animals at each sampling locations were observed and recorded. Vector collections were carried out using human landing catch and Mosquito Magnet (Model: Independence; Manufacturer: Woodstream Corp., USA)^17^. One Mosquito Magnet was used in this study. On the other hand, manual aspirator was used to collect the resting *Anopheles* mosquitoes. For mosquitoes collected using HLC, the mosquitoes were collected as soon as the mosquitoes landed before they bite. The mosquitoes were collected continuously between 1900 till 2300 h by two to three trained personnel each night for a total of 27 nights from all the sampling locations. All the mosquito collection using the three methods were carried out on the same time frame.

### Mosquito rearing

Laboratory strain *An. cracens* was used for all the laboratory-based experiments in this study. The eggs of self-mating colony of *An. cracens* were acquired from the Department of Parasitology laboratory, Chiang Mai University, Thailand. The eggs were transferred into a white larval rearing pan (20 × 30 × 5 cm) which had been half filled with dechlorinated water. Once the first instar larvae emerged, a sprinkle of finely grounded TetraBits Complete® food was added. On development, pupae were collected from the trays with a disposable pipette and transferred into a plastic container (17 × 11 × 6 cm) half filled with dechlorinated water. The container was then transferred into a 30 × 30 × 30 cm rearing cage (BugDorm-1 Insect Rearing Cage, Taiwan) until the adult mosquitoes emerged. The adult mosquitoes were fed with 10% sugar solution with vitamin B complex. The mosquito colony was maintained as described by Amir et al.^46^ and Andolina et al.^47^ in a secure insectarium maintained at 26 °C and relative humidity of 80% with alternating 12 h cycles of light and dark. To promote eggs production, few female mosquitoes were isolated in a cup and fed with human blood drawn in EDTA tube using Hemotek® membrane-feeding system.

### Laboratory assessment on *An. cracens* meal preference after the initial blood meal

**Figure 6:**
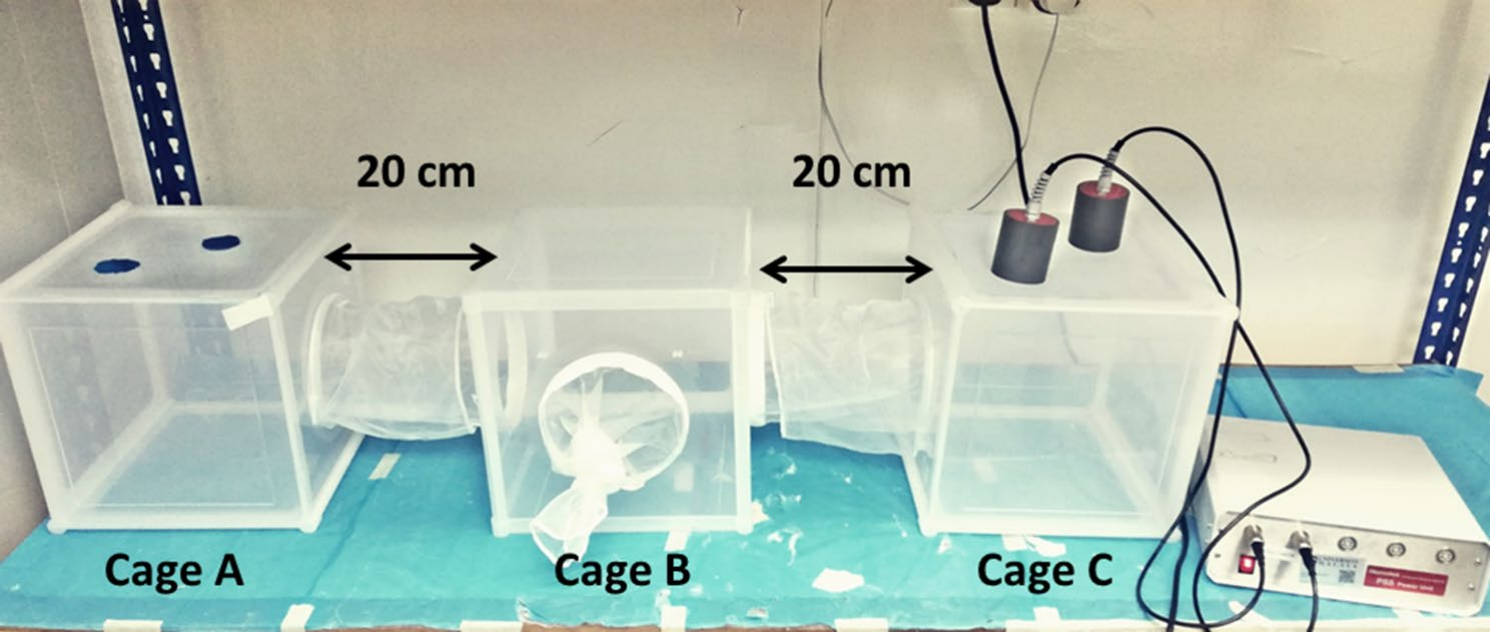
The triple cage setup to study the meal preference of the *An. cracens* mosquitoes.

A triple cage (Fig. 6) was used to study the response of blood-fed *An. cracens* mosquitoes to a second blood meal within a single gonotrophic cycle. Cage A was prepared with two cotton pads soaked with 10% sugar solution infused with one drop of Brilliant blue food dye^48^. The surface area of the cotton pad was prepared with the same size as the Hemotek membrane feeder which was 3.5 cm in diameter. On the other hand, two membrane feeders were prepared for Cage C. Human blood drawn in the EDTA tube was infused with 0.1% rhodamine B fluorescent marker (Sigma-Aldrich, USA)^49^ before transferring into the Hemotek membrane feeder. The sucrose cotton pads and blood meal were alternated between Cage A and C for six biological replicates to ensure there was no bias.

On day 0, a total of 75 fully engorged mosquitoes (8–12 days old) with human blood were carefully selected and separated into 3 different cups each containing 25 mosquitoes. The experiment was conducted for 3 days (the estimated duration of a single gonotrophic cycle) where each day one cup containing 25 mosquitoes was used to study the meal preferences. After 24 h post-feeding (Day1), the first cup containing the blood-fed mosquitoes were transferred to the holding chamber (Cage B) and allowed 20 min for the mosquitoes to acclimatize to the test cage environment. The openings at both sides of the holding chamber were closed to prevent the mosquitoes escaping to other cages before the experiment begins. Once the mosquitoes were rested, both sides of the cages were opened to allow the mosquitoes to roam freely to select its second meal. At the end of 3 h, the openings between the cages were closed and the mosquitoes were collected from each cage (A and C) into separate cups. Every mosquito was observed under both stereomicroscope (Olympus SZ) and fluorescent microscope with RFP2 filter (Olympus U-RX-T). If the mosquitoes had taken a second blood meal, it will fluoresce under the fluorescent microscope while if it had taken a sugar meal, the abdomen will appear blue under the stereomicroscope. Experiments were all performed between 0800 and 1200 h to avoid diurnal changes in mosquito activity^48^. The experiment was then repeated at 48 h (Day 2) and 72 h (Day 3) post feeding.

### Time limits for blood meal detection

The degree of blood meal digestion over a time course was evaluated through the amplification of host DNA ingested by *An. cracens* mosquitoes (8–12 days old). Mosquitoes were held for various time points post-feeding to determine how long the host DNA was detectable in extracted midgut of the mosquitoes using PCR assay. Only mosquitoes which had fully engorged were used in the experiment. Both human and macaques (*Macaca fascicularis*) blood were tested in this time course experiment. At every subsequent 12 h from the blood meal, five mosquitoes were collected in a small cup. The midgut of the mosquitoes was dissected under a stereomicroscope and stored in a 1.5 mL Eppendorf tube for genomic DNA extraction using the DNeasy tissue kit (Qiagen, Germany) according to the manufacturer’s protocol. The process was carried out for each host group through a time course of 96 h post feeding. The 0 h post feeding also acted as positive control where the mosquitoes were immediately killed after blood feeding. To prevent possible cross contamination with human DNA during the extraction process, the DNA of a male mosquito was extracted together with the other samples which acted as negative control. All the extracted DNA was later subjected to nested PCR as described below^50^. A total of 3 biological replicates for each host DNA were carried out.

### Multiple hosts feeding analysis

Laboratory based experiments were conducted to evaluate the ability of the PCR assay to detect multiple blood meal from different hosts. This experiment was carried out to simulate the actual scenario in the field where the mosquitoes might have taken multiple blood meals from different hosts in a single gonotrophic cycle. To mimic the transmission dynamic of knowlesi malaria, monkey and human blood were used in this experiment. A total of 75 *An. cracens* mosquitoes aged between 8 – 12 days were fully fed with monkey blood on Day 0. Then, 25 engorged mosquitoes were removed at day 1, day 2 and day 3 respectively and fed with human blood infused with 0.1% rhodamine B fluorescent marker (Sigma-Aldrich, USA)^49^ to identify the mosquitoes which had taken the human blood. The genomic DNA was extracted from the fluoresce mosquitoes on the same day and tested for the presence of both vertebrates’ DNA by animal specific PCR assay using the protocol described below^51^. A total of three biological replicates were carried out.

### Ethic approval

This study was approved by Medical Research and Ethics Committee, Ministry of Health Malaysia (NMRR-19-962-47606). All methods used in this study were performed in accordance with the relevant guidelines and regulations. The trained mosquito collectors were provided with antimalarial prophylaxis. The project also provided free blood examination on day 10 after mosquito collection. Informed consent was obtained from all participants.

### Collection of field *Anopheles* mosquitoes

**Figure 7:**
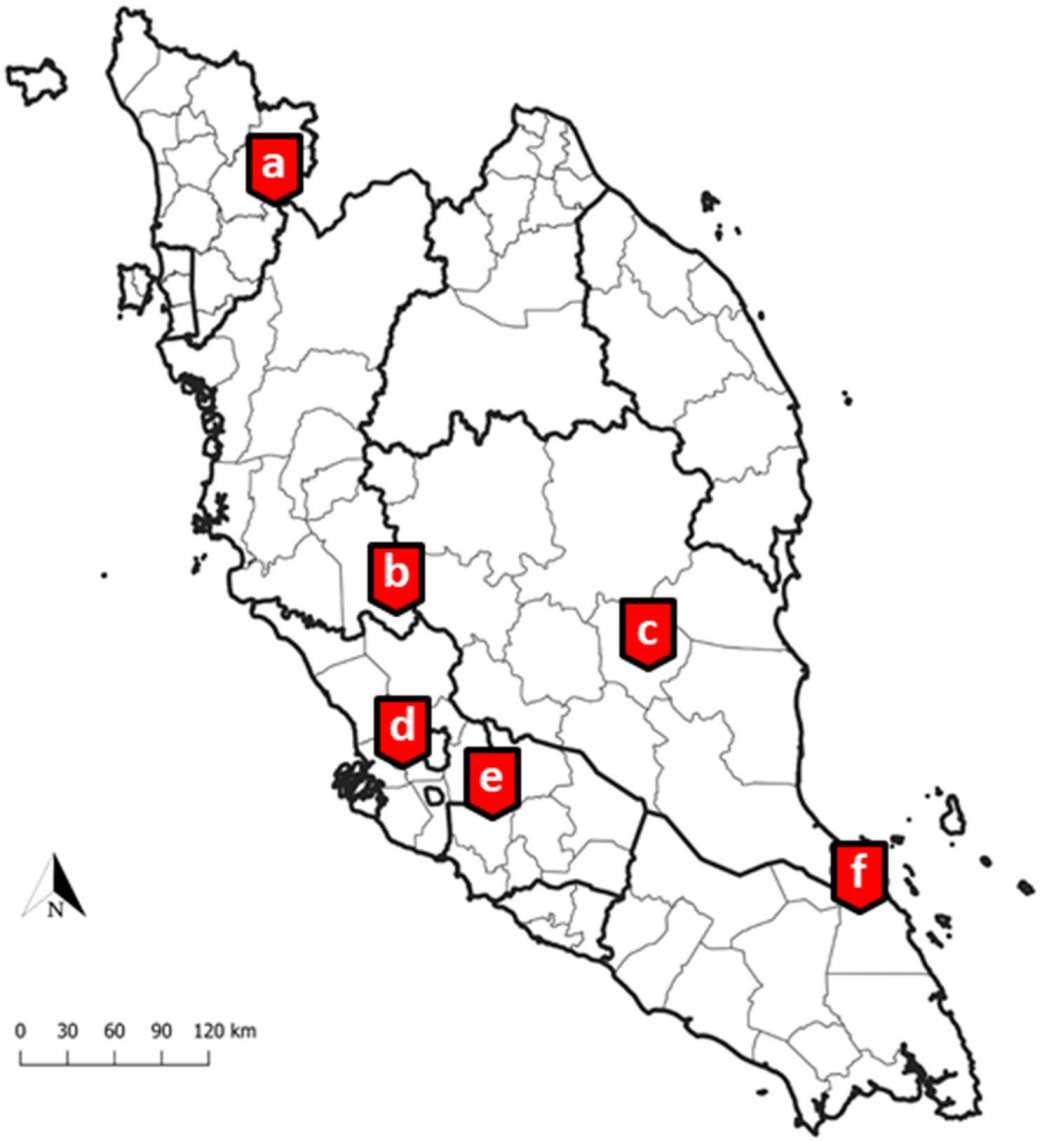
Map of Peninsular Malaysia where the mosquito collections were performed in six different states (**a**) Kampung Kaki Bukit, Baling, Kedah; (**b**) Sungai Dara, Muallim, Perak; (**c**) Kem Sri Gading, Jengka, Pahang, (**d**) Lenggeng forest, Seremban, Negeri Sembilan, (**e**) Community forest reserve, Kota Damansara, Selangor and (**f**) Bukit Tinggi, Mersing, Johor. The map was created using QGIS software version 3.6.3 (https://www.npackd.org>qgis64>3.6.3).

Adult female *Anopheles* mosquitoes were collected from six different states in Peninsular Malaysia: Johor, Kedah, Negeri Sembilan, Pahang, Perak and Selangor (Fig. 7). In Johor, forested area in Bukit Tinggi (2° 17′ 14.1″ N, 103° 40′ 27.8″ E) was selected as the study location. It is a virgin forest (less explored or exploited by human activity) situated on hilly terrain where an army camp was located at the hilltop. In Kedah, the sampling was conducted in Kampung Kaki Bukit, Baling (5° 42′ 16.4″ N, 100° 57′ 29.4″ E). It is a small village with scattered village houses located near to the forest fringes. Most villagers kept livestock such as chickens and bovines in their compound shed. Pets such as dogs and cats were also common animals sighted in this area. Most of the houses were made of wooden walls with aluminum rooftops and an open eaves for aerations. In Negeri Sembilan, the study was conducted in Lenggeng forest (2° 53′ 17.6″ N, 101° 57′ 24.9″ E). The study location was characterized by undulating hills and valleys with a few scattered aboriginal settlements. A large portion of the area is covered by oil palm plantation bordering to a secondary forest. Dogs and chickens are common animals seen near the aboriginal settlements. On the other hand, in Pahang, the camping site, Kem Sri Gading (3° 45′ 37.9″ N, 102° 34′ 20.2″ E) at Jengka was chosen as the sampling location. Besides being used as camping site, this forest is usually used for hiking or as a cycling trail. In Perak, the mosquito collection was carried out near Sungai Dara, Muallim (3° 47′ 46.6″ N, 101° 31′ 15.2″ E). Major portion of the sampling location were vegetated by secondary forest where some of the parts were undergoing deforestation. The sampling location was near to a river with potential mosquito breeding sites especially where the water pockets were formed. In Selangor, a community forest reserve in Kota Damansara (3° 10′ 06.0″ N, 101° 34′ 50.7″ E) was chosen as the sampling location. It is a 320 hectares dipterocarp rainforest in the midst of the urban sprawl. Presence of a number of trail networks make it an ideal recreational place for the surrounding urban dwellers. There are many monkeys and wild dogs sighted at this area. In all the sampling locations, human presences are inevitable. Besides, since all the sampling locations were either at forested area or forest fringes, macaques and wild boars were commonly sighted. In addition, there were also human knowlesi malaria cases reported within 2 km radius from all the sampling locations (except for Kota Damansara) between 2011 till 2019 (Unpublished data from Ministry of Health Malaysia).

### Identification of field *Anopheles* mosquitoes

The collected *Anopheles* mosquitoes were morphologically identified to the species level by using the taxonomic keys of Reid^37^ and Sallum^52^. However, for *Anopheles* mosquitoes from the Leucosphyrus Group and mosquitoes which were not able to be identified morphologically, the species were molecularly confirmed through DNA sequencing of the PCR amplified *ITS2* gene using ITS2A and ITS2B primers^53^ with protocol from the previous study^17^.

### Sample preparation and blood meal analysis

The abdomen of female *Anopheles* mosquitoes collected from the field were separated from the head and thorax under the dissecting microscope and were kept in 95% ethanol for genomic DNA extraction. The abdomen of the mosquitoes was homogenized and the DNA was extracted using the DNeasy tissue kit (Qiagen, Germany) according to the manufacturer’s protocol. The extracted DNA was subjected to nested PCR assay targeting vertebrate mitochondrial Cytochrome c Oxidase Subunit 1 (*COI*) gene to identify the presence of DNA from the vertebrate host. The primers and protocol used for identification of vertebrates’ DNA in the midgut of the mosquitoes were as described by Alcaide et al.^50^ with slight modifications. To confirm the species of the animal, the PCR products of few randomly picked positive samples from each animal groups were gel excised and sent for DNA sequencing.

PCR amplification reaction for nest 1 assay was performed in a final volume of 30 μL containing 5 μL of DNA template, 1 × Green GoTaq reaction buffer (Promega), 3.0 mM MgCl_2_ (Promega), 0.25 mM of dNTPs mixture (Promega), 0.16 μM each of forward (M13BC-FW) and reverse (BCV-RV1) primers and 1.25 U of GoTaq DNA polymerase (Promega). Cycling parameter for nest 1 consisted of 4 min initial denaturation at 94 °C, followed by 35 cycles of 94 °C for 40 s, 45 °C for 1 min, 72 °C for 1 min and a final extension step at 72 °C for 7 min. For each 30 μL of nest 2 reaction, 3 μL of nest 1 PCR amplification product was used as DNA template. The concentrations of reagents used in the nest 2 amplifications were identical to those used in the nest 1 reactions except the final concentration of the GoTaq DNA polymerase (Promega) is 1.0 U with the primers used were M13-FW and BCV-RV2. The PCR condition for nest 2 consisted of 3 min of initial denaturation at 94 °C followed by a touch down protocol decreasing the annealing temperature from 60 to 45 °C during 40 s (− 1 °C/ cycle), with 40 s denaturation at 94 °C and 1 min extension at 72 °C, followed by 24 cycles of 94 °C for 40 s, 45 °C for 40 s, 72 °C for 1 min and a final extension step at 72 °C for 7 min.

The samples which were positive for vertebrate’s DNA were subjected to species-specific PCR using the primers described by Gunathilaka et al.^51^. PCR amplifications were done in individual tubes for the detection of bovine, cat, chicken, dog, human, monkey and wild boar. PCR amplification reaction was performed in a final volume of 30 μL containing 3 μL of DNA template, 1 × Green GoTaq reaction buffer (Promega), 3.0 mM MgCl_2_ (Promega), 0.2 mM of dNTPs mixture (Promega), 0.25 μM each of forward and reverse primers and 1.25 U of GoTaq DNA polymerase (Promega). Cycling parameter consisted of 4 min initial denaturation at 94 °C, followed by 35 cycles of 94 °C for 30 s, 62 °C for 1 min, 72 °C for 1 min and a final extension step at 72 °C for 10 min.

### Data analysis

All the data were analyzed using statistical software SPSS version 25 (IBM, New York, USA). Normality of data distribution was evaluated by applying the Shapiro–Wilk test. Evaluation of significance of differences between the meal preference of *An. cracens* after initial blood feeding were analyzed using the non-parametric test, Kruskal–Wallis with Dunn’s post-hoc test of multiple comparison for the 3 groups: blood, sucrose and non-fed. Besides, one-way repeated measures ANOVA was employed to determine significance in the differences in the number of mosquitoes taken a second blood meal at 3 different time points after initial blood feeding. On the other hand, a chi-square test was used to assess whether collection methods of the *Anopheles* mosquitoes influence the success rate of identifying the blood meal of the mosquitoes. The level of statistical significance was set at *P* < 0.05 for all tests.

To demonstrate the anthropophilic nature of the field-caught *Anopheles* mosquitoes, human blood index (HBI) was calculated for each species collected using the following formula^54^.

$$Human\;blood\;Index = \frac{Number\;of\;mosquitoes\;which\;have\;fed\;on\;humans}{{Total\;number\;of\;mosquitoes\;whose\;blood\;meals\;have\;been\;identified}}.$$

Unfortunately, forage ratio and selection index to quantify host preferences were not calculated since a comprehensive host census at the study locations were not conducted; especially when some of the sampling locations were in deep forested area.

## Supplementary Information

Supplementary Tables.

Supplementary Figures.

## Data Availability

The authors declare that the data supporting the findings of this study are available within the paper and its supplementary information file (Supplementary Figs. S1–S3 and Supplementary Tables 1, 2).
