# Supplementary material for: Blood meal analysis of Anopheles vectors of simian malaria based on laboratory and field studies
Source: Sci Rep. 2022 Jan 10;12:354. doi: 10.1038/s41598-021-04106-w (PMC8748441; doi:10.1038/s41598-021-04106-w)
Supplement: Supplementary file 1 — Supplementary Tables. [file 41598_2021_4106_MOESM1_ESM.docx]

|  | HLC | | | MM | | | Resting mosquitoes | | |
| --- | --- | --- | --- | --- | --- | --- | --- | --- | --- |
| Total number of mosquitoes screened | 242 | | | 69 | | | 10 | | |
| Mosquitoes positive when screened using vertebrate's specific primers (%) | 108 (44.63) | | | 36 (52.17) | | | 8 (80.00) | | |
| Condition | Blood seen in abdomen | Blood not seen in abdomen | Unknown | Blood seen in abdomen | Blood not seen in abdomen | Unknown | Blood seen in abdomen | Blood not seen in abdomen | Unknown |
| Mosquitoes positive when screened using vertebrate's specific primers (%) | 34 (31.48) | 66 (61.11) | 8 (7.41) | 20 (55.56) | 9 (25.00) | 7 (19.44) | 8 (100.00) | 0 (0.00) | 0 (0.00) |
| Mosquitoes positive for human blood alone (%) | 14 (41.18) | 8 (12.12) | 1 (12.50) | 3 (15.00) | 3 (33.33) | 1 (14.29) | 4 (50.00) | 0 (0.00) | 0 (0.00) |
| Mosquitoes positive for mixed blood meal (with human blood) (%) | 16 (47.06) | 48 (72.73) | 5 (62.50) | 13 (65.00) | 5 (55.56) | 4 (57.14) | 4 (50.00) | 0 (0.00) | 0 (0.00) |
| Mosquitoes positive for vertebrate's DNA but negative for human blood (%) | 4 (11.76) | 9 (13.64) | 1 (12.50) | 3 (15.00) | 1 (11.11) | 2 (28.57) | 0 (0.00) | 0 (0.00) | 0 (0.00) |
| Animal host unidentified from the blood meal (%) | 0 (0.00) | 1 (1.52) | 1 (12.50) | 1 (5.00) | 0 (0.00) | 0 (0.00) | 0 (0.00) | 0 (0.00) | 0 (0.00) |

Unknown: The status of blood in the abdomen of the mosquitoes not recorded

**Supplementary Table S1:** Number and percentage of field caught *Anopheles* mosquitoes used in blood meal analysis according to the collection methods and also condition of the abdomen.

|  | Human | Chicken | Dog | Cat | Bovine | Monkey | Wild boar | HBI |
| --- | --- | --- | --- | --- | --- | --- | --- | --- |
| *An. aconitus* | 2 | 0 | 0 | 0 | 0 | 0 | 0 | - |
| *An. barbirostris* complex | 2 | 0 | 1 | 0 | 0 | 0 | 0 | - |
| *An. cracens* | 12 | 0 | 1 | 2 | 1 | 8 | 4 | 0.80 |
| *An. introlatus* | 22 | 0 | 1 | 1 | 0 | 13 | 9 | 0.76 |
| *An. maculatus* | 65 | 1 | 20 | 1 | 5 | 0 | 45 | 0.87 |
| *An. sinensis* | 26 | 0 | 20 | 0 | 0 | 0 | 19 | 0.90 |
| Total | 129 | 1 | 43 | 4 | 6 | 21 | 77 | 0.85 |

**Supplementary Table S2**: Number of mosquitoes according to the blood meal origins and Human Blood Index (HBI) of six *Anopheles* species from Peninsular Malaysia.
