# Supplementary material for: Blood meal analysis of Anopheles vectors of simian malaria based on laboratory and field studies
Source: Sci Rep. 2022 Jan 10;12:354. doi: 10.1038/s41598-021-04106-w (PMC8748441; doi:10.1038/s41598-021-04106-w)
Supplement: Supplementary file 2 — Supplementary Figures. [file 41598_2021_4106_MOESM2_ESM.pdf]

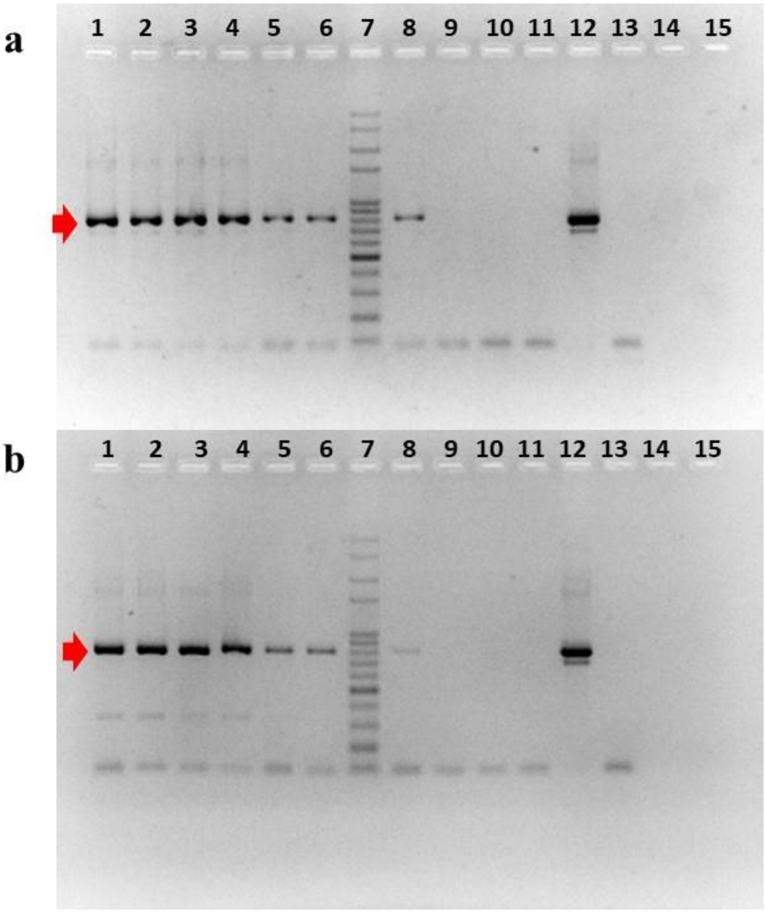

Supplementary Fig. S1: Agarose gel showing time course analysis for mosquitoes fed on (**a**) human blood and (**b**) monkey blood using vertebrate specific primers (800 bp). Lane 1, 0 hours post-feeding, Lane 2, 12 hours; Lane 3, 24 hours; Lane 4, 36 hours; Lane 5, 48 hours; Lane 6, 60 hours; Lane 7, 100 base pair plus ladder; Lane 8, 72 hours; Lane 9, 84 hours; Lane 10, 96 hours; Lane 11, negative control; Lane 12, positive control and Lane 13, Non-template control (NTC). Lane 14 and 15 are empty.

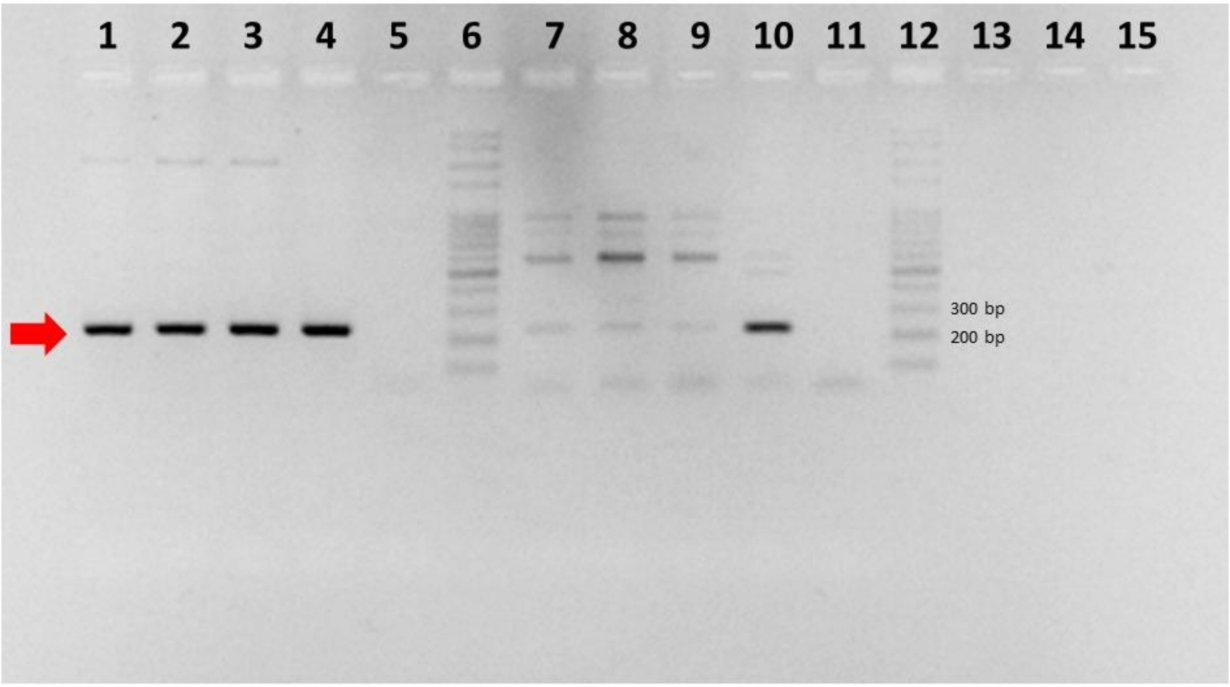

Supplementary Fig. S2: Agarose gel showing identification of mixed blood meal in *An. cracens*. Lane 1-5 showed amplified products using human specific primers (228 bp) while lane 7 – 11 showed amplified products using monkey specific primers (222 bp). Lane 1 – 3 showed amplified product of human blood post-feeding on day 1 until day 3. Lane 7 – 9 showed amplified product of monkey blood on day 1 until day 3 after feeding on day 0 (first blood meal). Lane 4, positive control for human blood while Lane 10, positive control for monkey blood. Lane 5, non-template control for human blood while Lane 11, non-template control for monkey blood. Lane 6 and 12, 100 base pair plus ladder. Lane 13, 14 and 15 are empty.

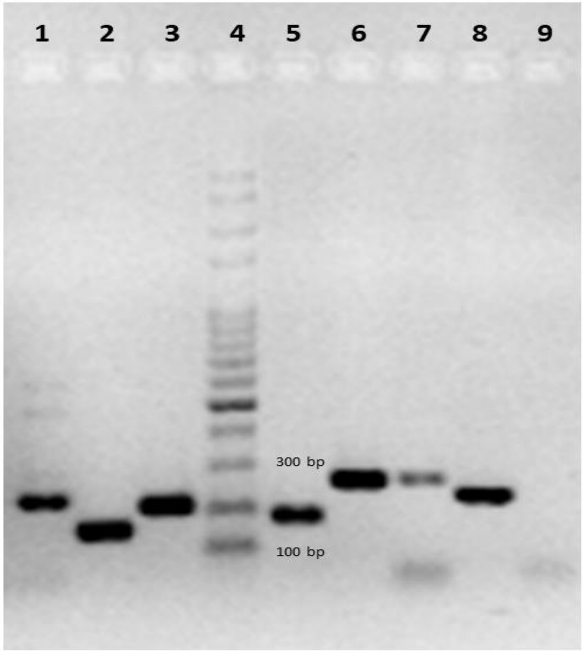

Supplementary Fig. S3: Agarose gel showing species specific Cytochrome c Oxidase Subunit 1 (*CO1*) gene amplified from host DNA using polymerase chain reaction (PCR). Control products amplified from host DNA are shown in Lane 1 (Monkey, 222 bp), Lane 2 (Dog, 153 bp), Lane 3 (Wild boar, 212 bp), Lane 5 (Cat, 180 bp), Lane 6 (Bovine, 271 bp), Lane 7 (Chicken 266 bp) and Lane 8 (Human, 228 bp). Lane 4 showed 100 base pair plus ladder while Lane 9 showed NTC (Non-template control).
